# Transesophageal Echocardiography During Cytoreductive Surgery with Hyperthermic Intraperitoneal Chemotherapy: A Novel Approach

**DOI:** 10.7759/cureus.5062

**Published:** 2019-07-02

**Authors:** Nasrin N Aldawoodi, Allan R Escher, David Ninan, Sephalie Y Patel

**Affiliations:** 1 Anesthesiology, H. Lee Moffitt Cancer Center and Research Institute, Tampa, USA; 2 Anesthesiology / Pain Medicine, H. Lee Moffitt Cancer Center and Research Institute, Tampa, USA; 3 Anesthesiology, Riverside University Health System Medical Center, Moreno Valley, USA

**Keywords:** tee, transesophageal echocardiography, hyperthermic intraperitoneal chemotherapy (hipec), cytoreductive surgery, euvolemia, fluid management, reduced ejection fraction, crs, cvp, novel approach

## Abstract

Cytoreductive surgery (CRS) with hyperthermic intraperitoneal chemotherapy (HIPEC) is an extensive, lengthy procedure for patients with peritoneal metastases. It is associated with fairly high morbidity and mortality as compared with other non-vascular intra-abdominal surgeries. Fluid and hemodynamic management is challenging and not well established, particularly in patients with a low ejection fraction (EF). This case details the successful intraoperative anesthetic management of a patient with an ejection fraction of 20% undergoing CRS/HIPEC using the addition of intraoperative transesophageal echocardiography (TEE) as an adjunct to central venous pressure (CVP), urine output (UOP), and calculated stroke volume variation (SVV) for goal-directed resuscitation and blood pressure support.

## Introduction

The combination of cytoreductive surgery (CRS) and hyperthermic intraperitoneal chemotherapy (HIPEC) is an aggressive procedure for cancer patients with peritoneal metastases. The procedure consists of two stages. First, all visible tumors are resected, as well as any involved intra-abdominal organs, over many hours through a laparotomy incision. Then, about one hour before the second stage, the patient is gradually cooled to < 35.5 °C to allow for the upcoming hyperthermia exposure. During the second stage, chemotherapy is infused directly into the abdominal cavity at 41-42 °C and the abdomen is manually shaken to distribute it for up to 120 minutes. On average, it is a nine-hour-long procedure, with insensible fluid losses, fluid shifts, acidosis, hypermetabolism, and hemodynamic perturbations [1]. Moreover, a certain amount of urine output (UOP) is targeted due to the nephrotoxicity of these chemotherapeutic agents, often necessitating mannitol or furosemide. Because this surgery is so extensive, studies have cited a morbidity rate of up to 50% [1-4]. The hyperthermia-induced hypermetabolic state is characterized by an increase in heart rate (HR), cardiac output (CO), central venous pressure (CVP), and pulmonary capillary wedge pressure (PCWP) as well as decreased systemic vascular resistance (SVR) and decreased effective circulating volume [5-6].

## Case presentation

The patient is a 69-year-old, 55 kg, 153 cm female with metastatic appendiceal adenocarcinoma who presented with an enlarging abdomen consistent with loculated, mucinous ascites and omental thickening. The preoperative computerized tomography (CT) scan of the abdomen and pelvis showed a large cystic mass in the pelvis associated with a soft-tissue element of the mass. It extended into the retroperitoneum and encapsulated the right renal artery, impressing on the posterior wall of the inferior vena cava. A moderate to large hiatal hernia was also noted. CRS/HIPEC surgery was recommended.

Other significant past medical history included stable gastroesophageal reflux disease (GERD) and a history of left-sided breast cancer status post-mastectomy and vertical rectus abdominis musculocutaneous (VRAM) flap. During her preoperative workup, the patient endorsed increasing bilateral lower extremity edema, abdominal distension and bloating. An electrocardiogram (ECG) also revealed q waves in the anterolateral leads; as a result, she was referred for cardiac evaluation. An echocardiogram revealed an EF of 20-25% with diffuse global hypokinesis. A left heart catheterization revealed no obstructive disease. Therefore, she was diagnosed as having chronic heart failure with reduced ejection fraction due to non-ischemic cardiomyopathy. The etiology was presumed to be from previous chemotherapy for breast cancer. She was started preoperatively on lisinopril and furosemide which led to a notable improvement in symptoms including resolution of orthopnea and decreased lower extremity edema. Her preoperative hemoglobin was 10.3 grams per deciliter (g/dL) and sodium was 133 milliequivalents per liter (L). All other preoperative labs were generally unremarkable. She did not have an automated implantable cardioverter defibrillator (AICD) or history of noted arrhythmias.

**Figure 1:**
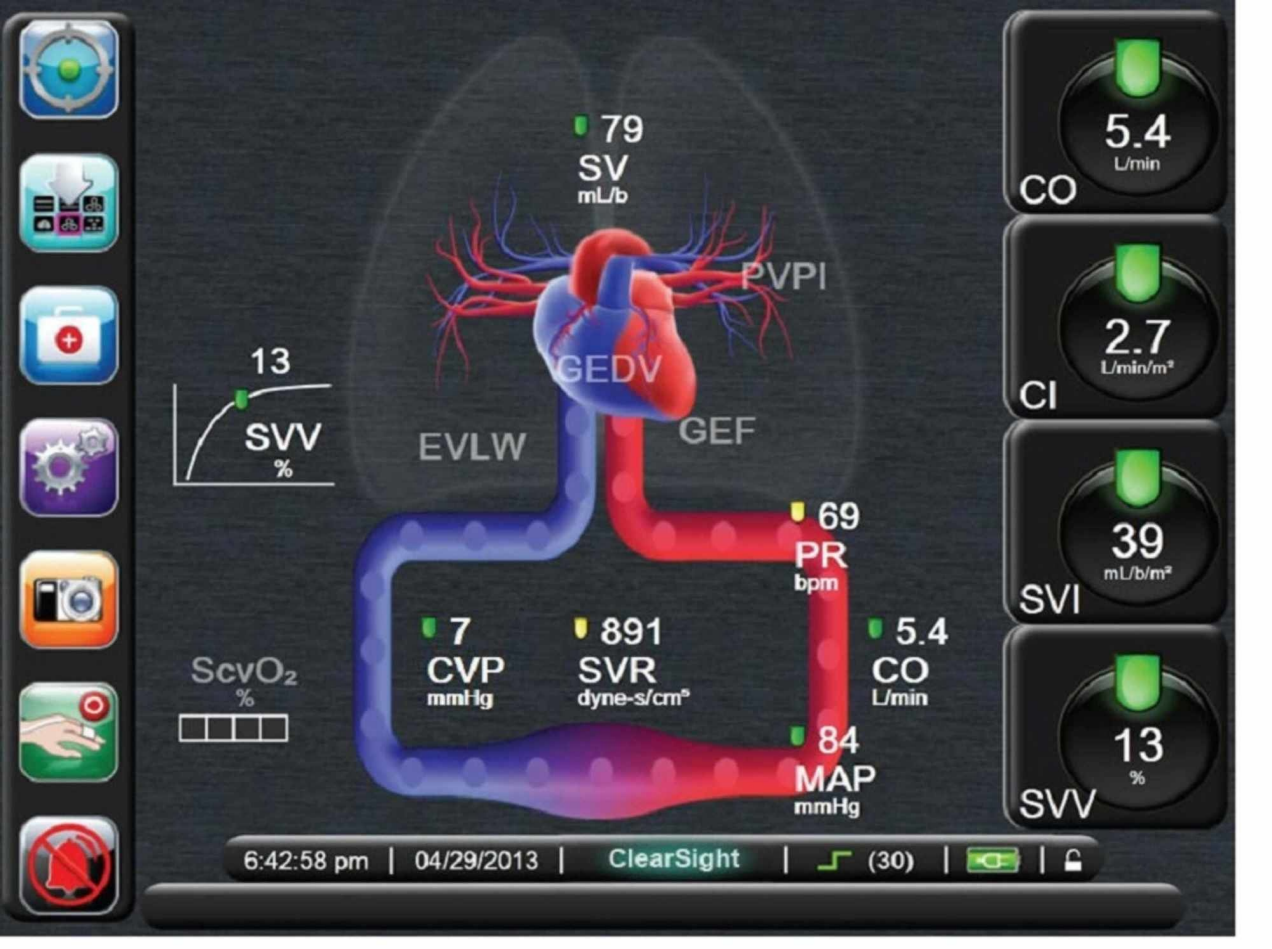
Flotrac/Vigileo system using data from arterial line and central venous line to calculate various hemodynamic parameters (stock image taken from Edwards.com) SVV - stroke volume variation; SV - Stroke Volume; SVI - stroke volume index; CVP - central venous pressure; CO - cardiac output; CI - cardiac index; PR - pulse rate; MAP - mean arterial pressure; SVR - systemic vascular resistance; GEDV - global end diastolic volume; PVPI - pulmonary vascular permeability index; EVLW - extravascular lung water; GEF - global ejection fraction

On the day of surgery, we decided to forego epidural placement, which is standard, for this case because we wanted fewer confounders in the workup of any post-operative hypotension. After applying standard American Society of Anesthesiologists (ASA) monitors and giving 2 mg intravenous (IV) midazolam, a pre-induction arterial line was placed. The patient was then pre-oxygenated and induced with 100 mcg IV fentanyl, 60 mg 2% IV lidocaine, 14 mg IV etomidate, and 80 mg IV succinylcholine. A 16-gauge peripheral IV catheter was placed and additionally, an 8 French (Fr), double lumen, 16 cm central venous cannula was placed in the right internal jugular vein under ultrasound guidance. Both the arterial line and central line were connected to a FloTrac/Vigileo device (Edwards Lifesciences, Irvine, CA, USA) which then calculated cardiac index (CI), systemic vascular resistance (SVR), stroke volume variation (SVV), and CVP (Figure 1).

**Figure 2:**
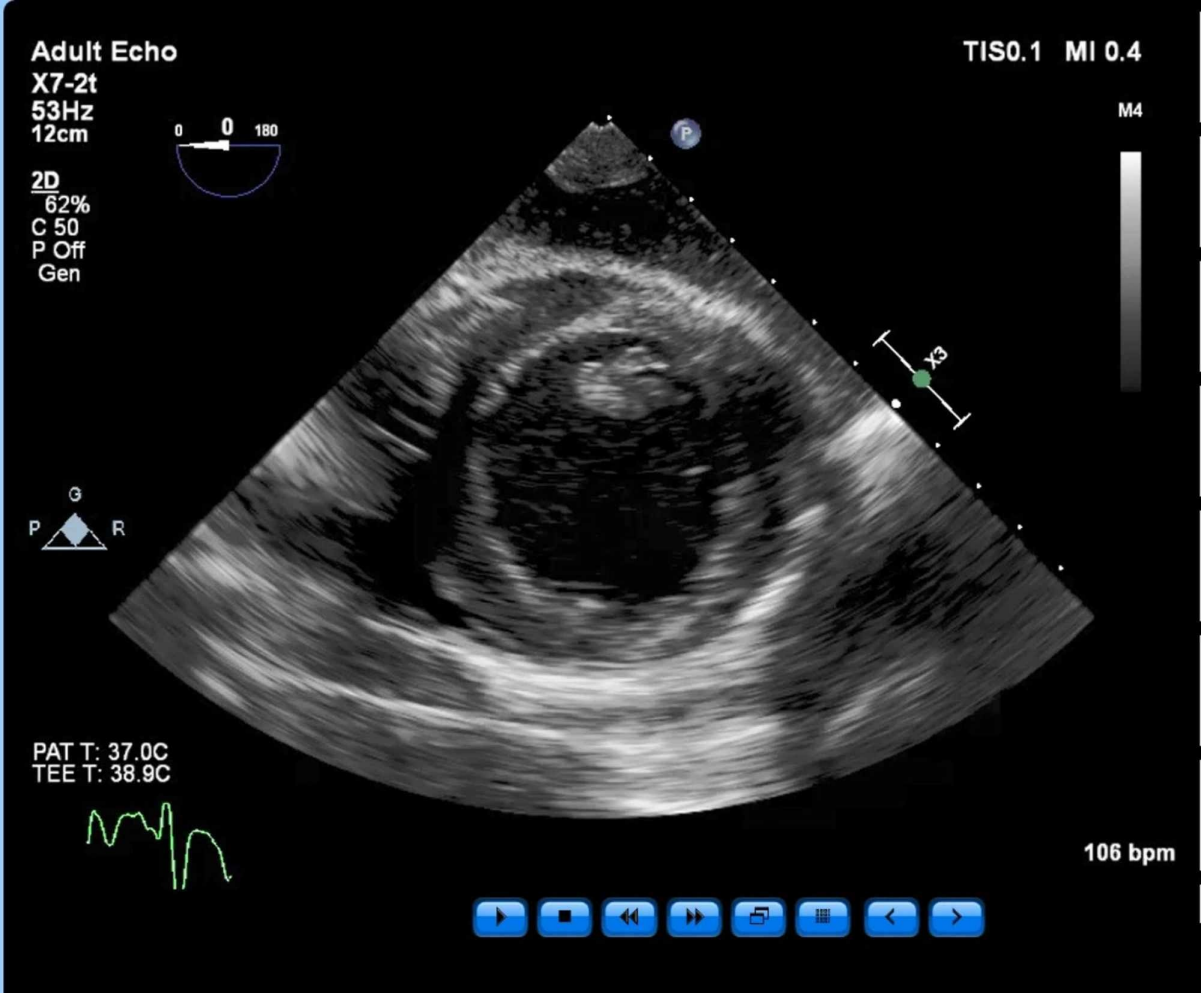
Baseline transgastric short axis view of the left ventricle T - temperature; bpm - beats per minute

We followed the trend of these numbers to guide fluid therapy. The initial values were CVP 17 mmHg, SVR 1500 dyn/s/cm^5^, CI 2.1, and SVV 3. A transesophageal echocardiography (TEE) probe was placed and connected to a Philips machine (Philips Professional Healthcare, Netherlands, Amsterdam). We performed a standard 28 view baseline exam (Figure 2).

**Figure 3:**
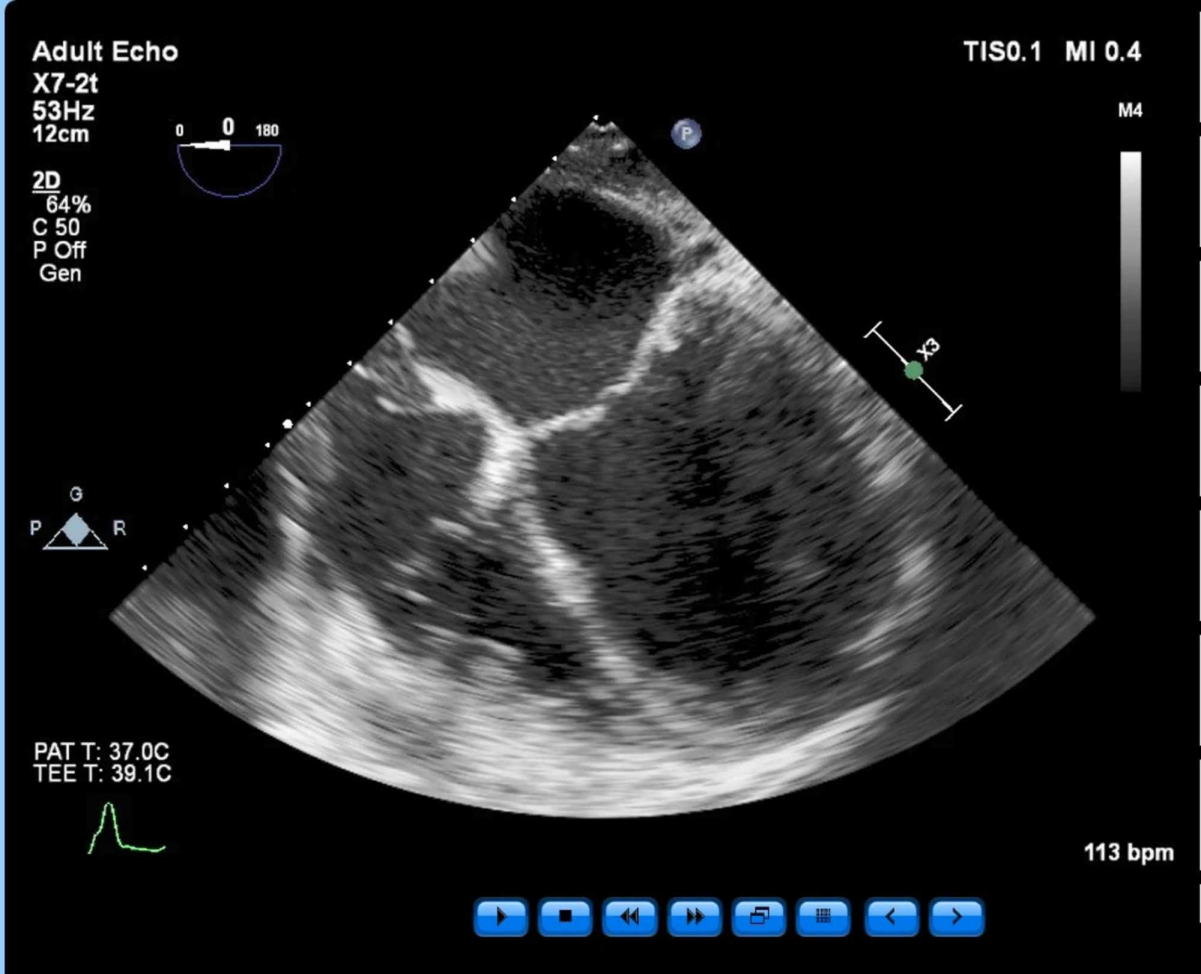
Mid-esophageal four-chamber view. Normal RV function was determined from this view. RV - right ventricle; T - temperature; bpm - beats per minute;

**Figure 4:**
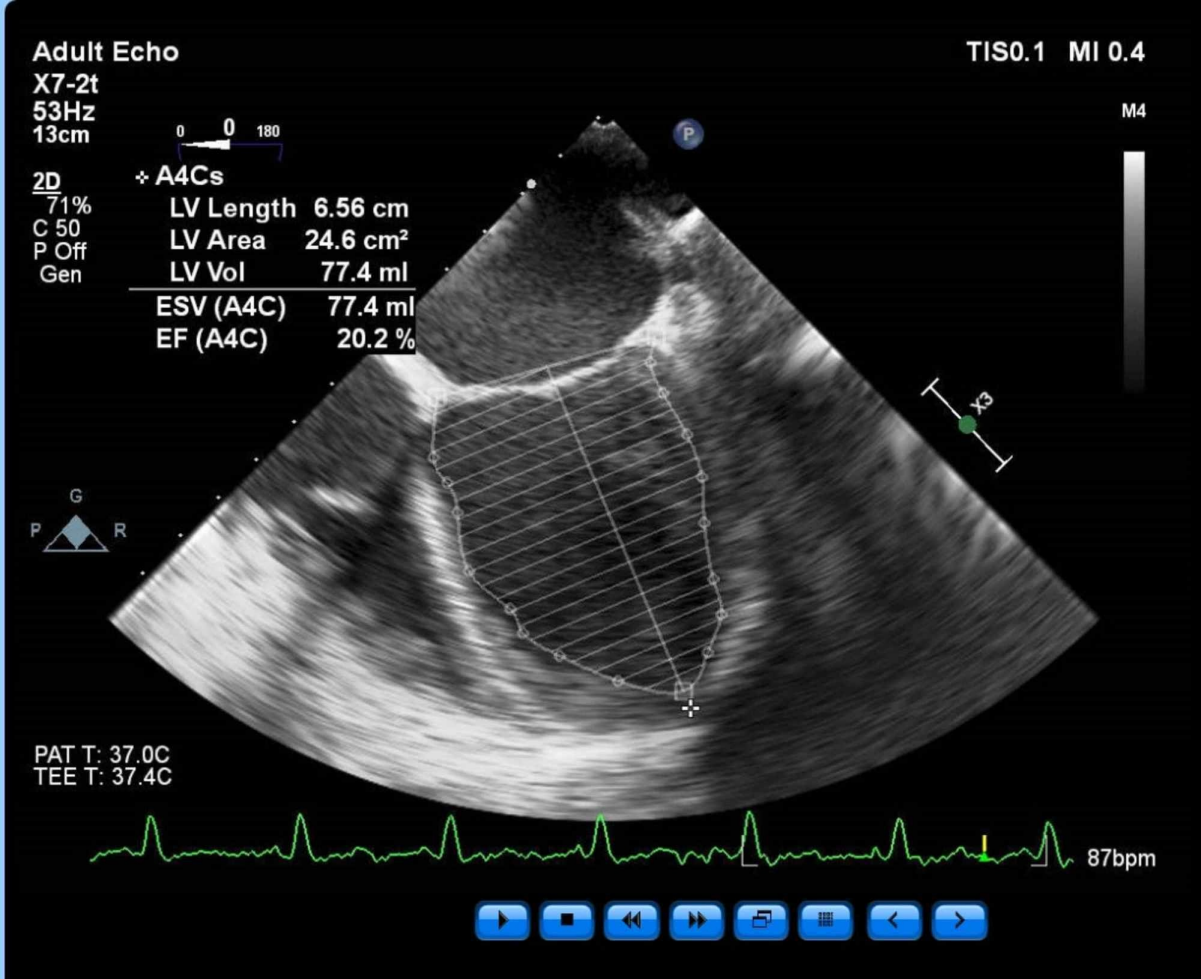
Left ventricle ejection fraction as calculated by Simpson’s Method. T - temperature; bpm - beats per minute

Findings included an ejection fraction (EF) of 25% and severe global hypokinesis as well as dilated left ventricle (LV) chamber, mild to moderate mitral regurgitation, no other significant valvular pathology, and normal right ventricle (RV) function (Figures 3-4). 

**Figure 5:**
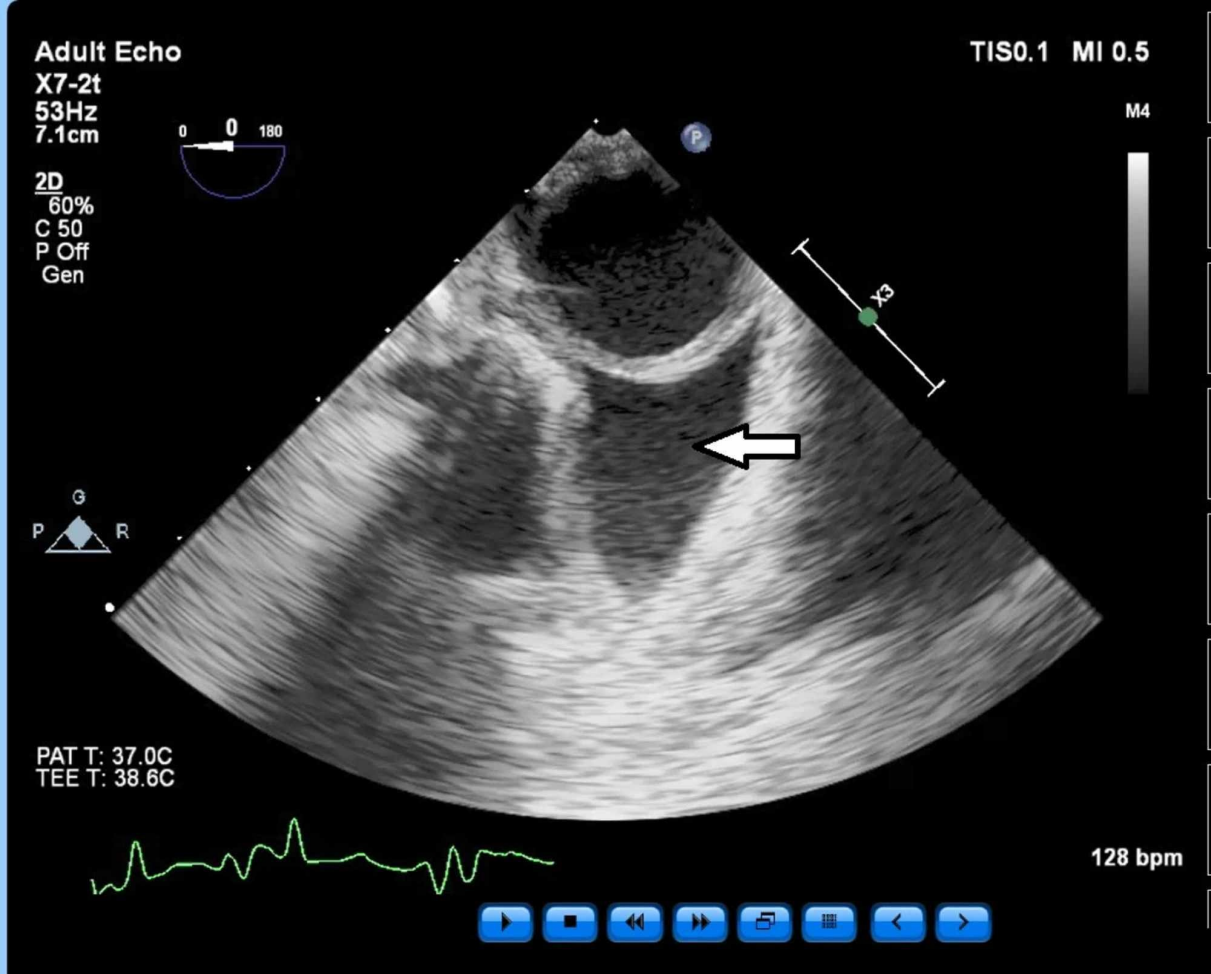
Short axis view of descending thoracic aorta showing incidental moderate left pleural effusion (arrow). T - temperature; bpm - beats per minute

The patient also was found to have a baseline left mild to moderate pleural effusion (Figure 5).

Premixed norepinephrine and epinephrine infusions were present in the room in case of any prolonged hypotension. Our plan was to be very judicious with fluids, making sure to keep our hands on the roller clamp any time medications were bolused, in order to immediately shut off the IV to avoid excessive crystalloid administration. We would preferentially use 5% albumin for volume if necessary and use SVV trend, CVP trend, UOP and TEE guidance during periods of hypotension, and periodically throughout the case to guide whether to administer colloid or vasopressor. Our vasopressor of choice to bolus was ephedrine due to inotropic qualities, and norepinephrine if we needed an infusion because of some Beta 1 activity providing inotropy without causing as much tachycardia and arrhythmia potential as other inotropes such as epinephrine and dobutamine. The patient’s baseline systolic blood pressure (SBP) was 114 mmHg; our goal was to stay within 20% which was an SBP > 90 mmHg. Our goal urine output was 0.5 mL/kg/hr which was about 30 mL/hr in this patient. We did not have a target amount of fluid replacement per hour and were rather going to give as little fluid as possible to maintain continued urine output and hemodynamic stability without high dose pressors. An SVV consistently greater than 13 along with hypotension was the threshold to give albumin or CVP continuing to trend down. We also looked at the trans-gastric short axis view of the left ventricle on TEE frequently to look for signs of the ventricular function worsening or improving with fluid or signs of the ventricle further dilating to signal possible fluid overload. 

The surgeon commenced by making a large laparotomy incision and immediately drained 15 L of mucinous ascites from the patient’s very large, protuberant abdomen. He then performed cytoreductive surgery which included: appendectomy, splenectomy, bilateral removal of adnexal tumor, including ovary and tube, resection of umbilicus, resection of the falciform ligament with tumor and peritoneal resections of the right flank (20 cm), pelvis (10 cm), right upper quadrant (20 cm), and left upper quadrant (10 cm). 

**Figure 6:**
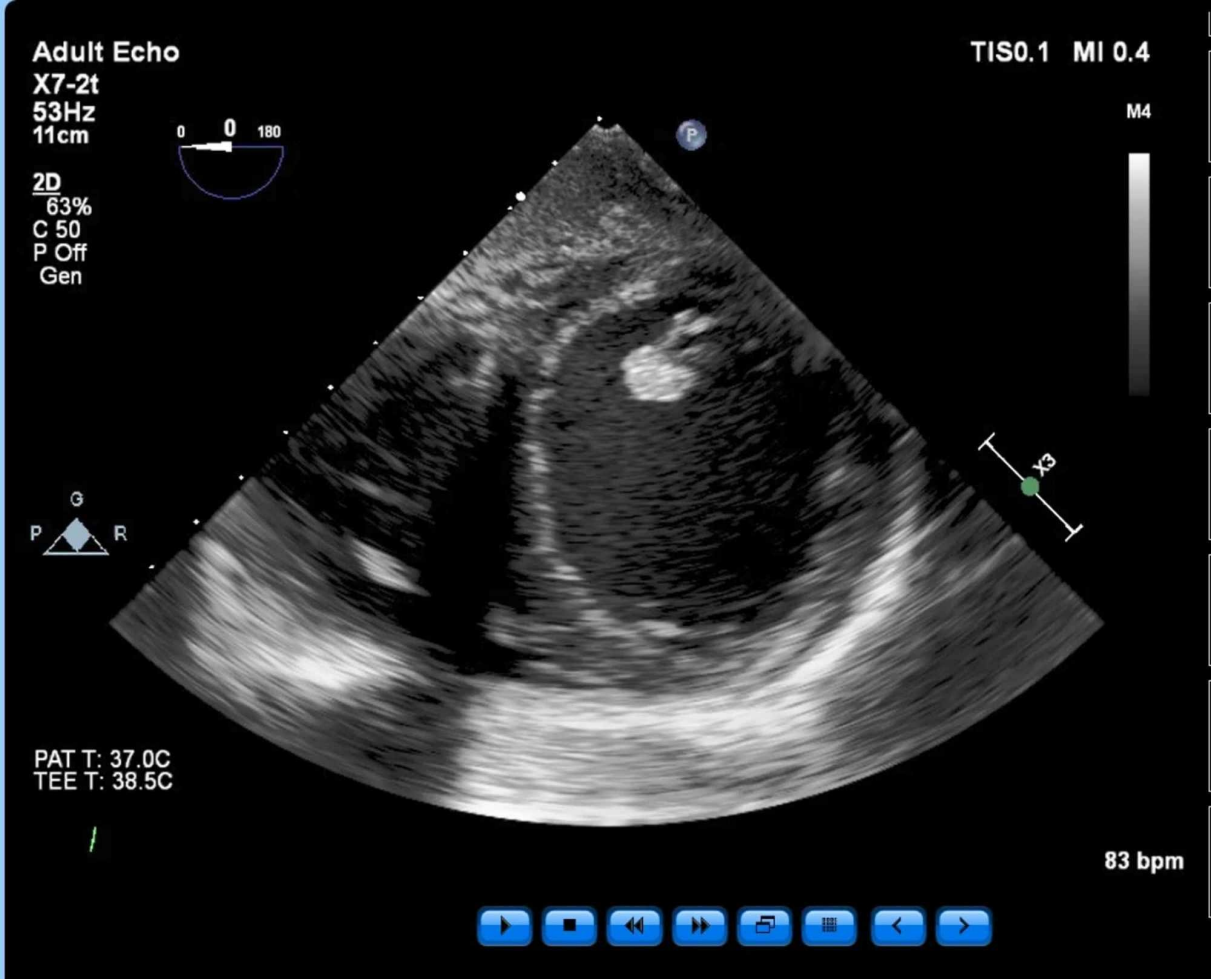
Transgastric short axis view of left ventricle showing ventricle appearing more dilated than baseline after fluid loading patient. T - temperature; bpm - beats per minute

After draining the initial 15 L of ascites, the CVP dropped from 17 mmHg to 8 mmHg. We examined the LV on TEE and the contractility appeared to improve, going from EF 25% to about 30%. This also corresponded to improvement in CI from 2.1 to 2.5. The SVV stayed the same (around 3) as did the SBP, so therefore we did not bolus any additional fluid during this time. About two hours into the surgery, we began to very slowly infuse 5% albumin as SVV was slowly increasing up to 15, the UOP had slightly dropped, and we were giving frequent bolus doses of ephedrine and phenylephrine. The left ventricle (LV) chamber appeared less dilated than baseline indicating possible fluid responsiveness. As we infused about 750 mL of 5% albumin over the course of three hours and a small bolus dose of crystalloid, SVV trended down to below 13 and the LV did appear more distended with somewhat decreased LV function and dyskinetic movement (Figure 6).

The CVP was unchanged over those few hours, but we decided based on the TEE that the patient was volume replete and we should stop our fluid infusion to avoid overload. 

After about six hours, the patient’s incision was closed and the HIPEC portion commenced. Inflow and outflow cannulas were inserted into the abdominal wall and the circuit was run with inflow at 43 °C and the outflow of approximately 41 °C to instill mitomycin. The patient was manually agitated during the perfusion. During the HIPEC portion, the patient had relative hypotension and tachycardia due to hypermetabolism from hyperthermia. Due to the mechanical agitation of the patient, our Flotrac/Vigileo numbers were not accurate so we periodically examined the TEE to confirm the stability of cardiac function and chamber size. Afterward, the patient’s abdomen was irrigated and hemostasis and closure was achieved. We checked the arterial blood gas (ABG) at three points during the case. Hemoglobin on the ABGs trended from 8.8 g/dL to 8.2 g/dL mid-case to 6.9 g/dL by the end of the case. A complete blood count (CBC) was sent which showed a hemoglobin of 7.3 g/dL and we decided not to transfuse as the patient was hemodynamically stable without vasopressors and mildly fluid overloaded. The base balance on ABG went from 0.7 to 2 to -3.6 immediately prior to extubation at the end of case and bicarbonate went from 27 to 21 from the beginning to the end of the case. The patient was never significantly acidotic. Electrolytes were checked midway through the case and creatinine was 0.5. Total surgical time and total anesthesia time were 8 hours, 46 minutes and 9 hours, 15 minutes, respectively.

**Figure 7:**
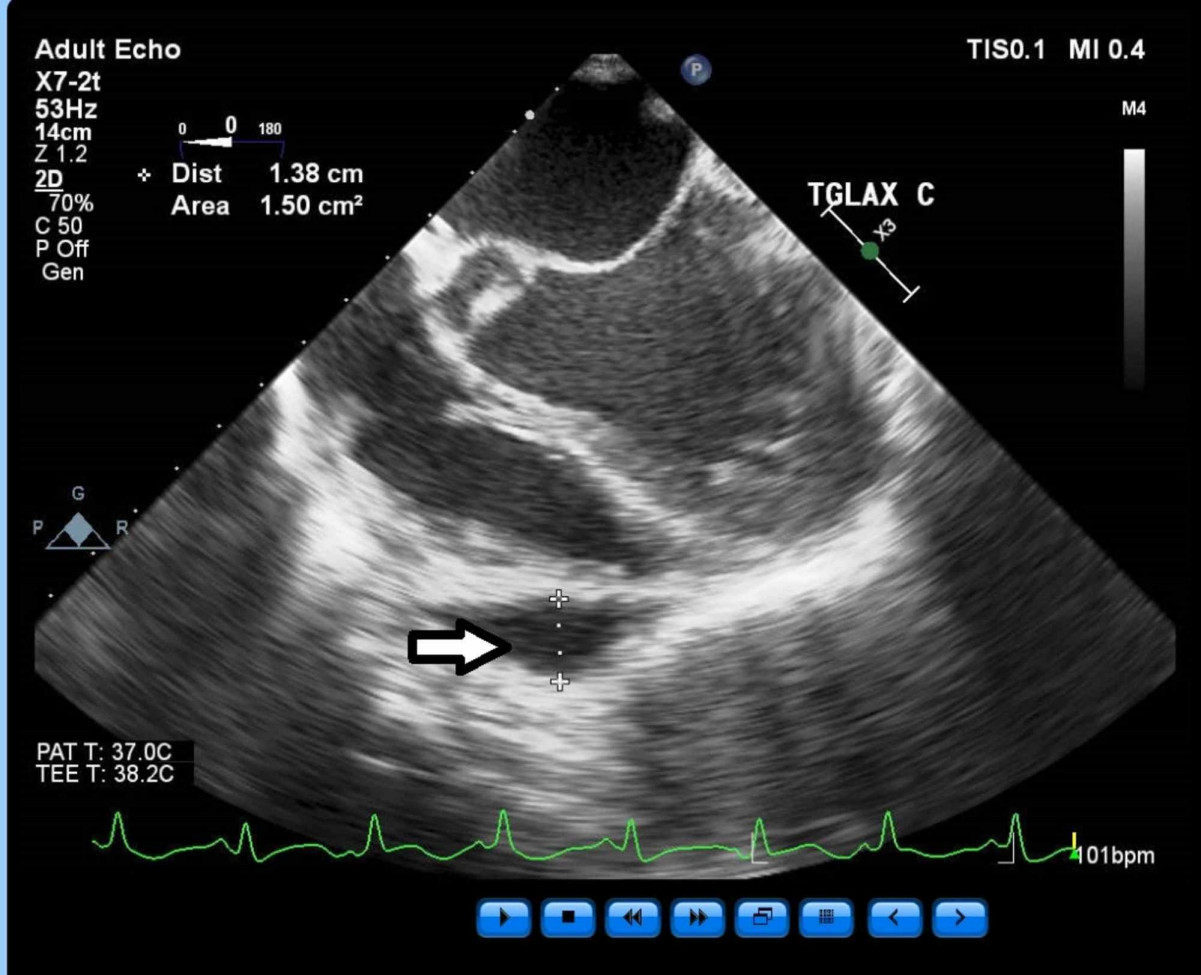
Pericardial effusion noted around the free wall of right ventricle at the end of the case, not hemodynamically significant (arrow). T - temperature; bpm - beats per minute

Regarding total fluid balance, the estimated blood loss was 150 mL and total urine output was 510 mL. We used a total of 1.7 L of crystalloid and 750 mL of 5% albumin. The hemodynamic parameters at the end of the case were a CVP of 6, SVV of 7, and TEE showed the LV function and chamber size was back to the patient’s beginning of surgery baseline. There were no new wall motion abnormalities and there was interval development of a small pericardial effusion without hemodynamic consequence to which we alerted the surgeon (Figure 7).

The left pleural effusion was stable to slightly enlarged at the end of the case.

The patient was extubated at the end of the case and taken to the post-anesthesia care unit (PACU) with stable vital signs. Her post-operative course showed consistent improvement with each day, no requirement for supplemental oxygen, no signs or symptoms of gross fluid overload or heart failure, and excellent kidney function. Her prescription for lisinopril and furosemide was restarted post-operatively. The patient was discharged from the hospital to her home on post-operative day eight (POD) in stable and good condition.

## Discussion

Fluid administration and maintenance of euvolemia can be an anesthetic challenge. As per one study which looked at 335 cases, the median total volume of fluid administered intraoperatively was 11 liters [1]. Intraoperative under-resuscitation and hypovolemia is an established risk factor for complications [2]. However, excessive fluid administration has also been linked to adverse outcomes in CRS/HIPEC [1]. In patients with preserved cardiac function, an ideal strategy for fluid management has yet to be clearly established. Furthermore, evaluating fluid status in HIPEC is made more difficult due to factors such as patient fasting, administering epidural analgesia, fluid shifts, and subcutaneous fluid accumulation, blood loss, draining of large volume ascites, and insensible losses. Measuring urine output (UOP) as an indicator of fluid status during HIPEC can also be unreliable as the patient often receives inotropes and diuretics to augment urine output and minimize nephrotoxicity [2]. Other studies have also found CVP to be an inaccurate predictor of volume status in CRS/HIPEC due to changes in intra-abdominal pressure and patient positioning during the procedure [7].

The literature discusses multiple methods to help guide goal-directed intraoperative therapy with a pulmonary artery catheter serving as a gold standard for preload, afterload, and cardiac output data. However, a pulmonary artery (PA) catheter can be associated with a risk of up to 5% of complications such as pneumothorax, arrhythmias, and PA perforation and has not been shown to be clearly beneficial to patient outcomes [2]. The FloTrac/Vigileo machine displays a non-invasive technique, utilizing an arterial line to measure the area under the blood pressure (BP) curve when combined with patient data input and also provides the stroke volume (SV), cardiac output (CO), and stroke volume variation (SVV) [2]. SVV > 13 is used as a number cutoff for hypovolemia at which point any hypotension should likely be treated with a fluid bolus. The data is mixed on the utility of Flotrac/Vigileo in CRS/HIPEC with some studies showing neither any significant restriction of fluid administration with its use versus standard techniques (arterial line and CVP) nor improvement in the clinical course [2]. Other studies have found the use of non-invasive SVV monitors such as Flotrac/Vigileo or LiDCOrapid (LiDCO, London, UK) results in greater intraoperative hemodynamic stability and a lower incidence of postoperative end-organ complications, corresponding with a decrease in measured lactate at the end of the procedure [8-9].

There are no current studies looking specifically at intraoperative transesophageal echocardiography to guide hemodynamics and fluid management in HIPEC/CRS. There is also minimal data on the management of patients with concomitant heart failure and severely reduced ejection fraction (EF< 30%) undergoing this procedure. In fact, most studies eliminated this patient cohort entirely from data analysis [6]. The most frequently cited goal of 12 milliliters per kilogram per hour (mL/kg/hr) fluid replacement for this procedure would seem too liberal for a patient with an EF of 20% [6]. In a recent study looking at over 600,000 patients undergoing non-cardiac surgery, it was found that patient with a diagnosis of heart failure had a higher risk of 90-day postoperative mortality, even in the patient subset with preserved ventricular function. Furthermore, the risk of postoperative mortality progressively increased with decreasing systolic function [10]. 

## Conclusions

Our case is unique in the literature, as it discusses the successful intraoperative anesthetic management of a patient with an EF of 20%, presenting for CRS/HIPEC using intraoperative transesophageal echocardiography as a supplement to CVP, non-invasive SVV monitoring, and UOP to closely guide management of hemodynamics and fluid replacement. 
